# Cross-Field Road Markings Detection Based on Inverse Perspective Mapping

**DOI:** 10.3390/s24248080

**Published:** 2024-12-18

**Authors:** Eric Hsueh-Chan Lu, Yi-Chun Hsieh

**Affiliations:** Department of Geomatics, National Cheng Kung University, No. 1, University Rd., Tainan 701, Taiwan; situn50627@gmail.com

**Keywords:** road markings, object detection, cross-field, inverse perspective mapping, deep learning

## Abstract

With the rapid development of the autonomous vehicles industry, there has been a dramatic proliferation of research concerned with related works, where road markings detection is an important issue. When there is no public open data in a field, we must collect road markings data and label them by ourselves manually, which is huge labor work and takes lots of time. Moreover, object detection often encounters the problem of small object detection. The detection accuracy often decreases when the detection distance increases. This is primarily because distant objects on the road take up few pixels in the image and object scales vary depending on different distances and perspectives. For the sake of solving the issues mentioned above, this paper utilizes a virtual dataset and open dataset to train the object detection model and cross-field testing in the field of Taiwan roads. In order to make the model more robust and stable, the data augmentation method is employed to generate more data. Therefore, the data are increased through the data augmentation method and homography transformation of images in the limited dataset. Additionally, Inverse Perspective Mapping is performed on the input images to transform them into the bird’s eye view, which solves the “small objects at far distance” problem and the “perspective distortion of objects” problem so that the model can clearly recognize the objects on the road. The model testing on the front-view images and bird’s eye view images also shows a remarkable improvement of accuracy by 18.62%.

## 1. Introduction

With the rapid development of the autonomous vehicles industry, there has been a dramatic proliferation of research concerned with related works. For the purpose of driving the car automatically, autonomous cars must be able to perceive the surrounding environment at any time and handle emergency conditions, so it is essential that detecting and classifying objects accurately in the road environment will ensure the stability of the driving. Not only have autonomous vehicles gained considerable attention but High-Definition Maps have also aroused wide concern. High-Definition Maps are maps for autonomous vehicles that contain a great deal of information about the road environment, such as road boundaries, road lanes, traffic signs, road markings, etc., so that the computing platform inside the vehicle can construct spatial awareness and provide assistance for the autonomous vehicles’ decision-making systems. The High-Definition Maps are composed of point cloud layers and vector layers that conform to the attribute content defined by the High-Definition Maps standard. In order to ensure the quality and consistency of High-Definition Maps, many procedures are often labor-intensive and time-consuming, especially the production of semantic feature maps (vector layers). To improve the performance of semantic feature extraction for High-Definition Maps, object detection based on Convolutional Neural Networks (CNNs) for automatic extraction of semantic features has become very common over the years. The purpose is not only to recognize and classify the objects but also to indicate the relative position of the objects in the image. According to the format standards of High-Definition Maps, the vector layers consist of the actual shape of the road markings as a polygon. The research is based on establishing the geometric outline objects of the High-Definition Map to set the pixel-level road markings detection.

In order to recognize the objects precisely, the Artificial Intelligence (AI) decision model needs to learn a large amount of image data that include different weather conditions to improve the robustness of the model. However, data collection and labeling data cost human resources and time; thus, an efficient data collection method should be determined. Previous studies on the detection of objects mostly focus on objects on the upper part of the road such as road signs and traffic signals, and less on road markings on the road surface. Since the detection targets are located on the road, only the area of the road environment is required. Generally, this kind of research will carry out image processing in the preprocessing stage. Common practices removing irrelevant backgrounds include extracting regions of interest, front view to bird’s eye view, and so on, which can reduce the influence of environmental conditions and unnecessary background feature learning so that the deep-learning model can achieve better performance.

Image data with ground truth is an important key for determining the accuracy of object detection. If the cost of data labeling can be reduced, high-quality road image recognition and application can be improved. However, data collection takes a long time and costs a large amount of money. For instance, an intelligent vehicle equipped with industrial cameras in the field where the route has been surveyed and planned acquires the data for model training. After data collection, the self-collected data need to be labeled manually, which is huge additional labor work and takes a large quantity of time. Furthermore, there is no public road markings dataset in Taiwan causing difficulties in the use of data for related research. Additionally, one of the most crucial tasks for autonomous vehicles is to detect the lane and road markings accurately. In current methods, when the detection distance increases, the detection accuracy of objects frequently declines. This is primarily because distant objects on the road take up few pixels in the image and because object scales vary depending on different distances and perspectives. In light of these concerns, this paper expects to solve the problem of reduced detection accuracy resulting from small objects and the difficulties of data collection.

To solve the problems mentioned above, the research utilizes a virtual dataset and an open dataset to train the object detection model and cross-field testing in the field of Taiwan roads without spending a lot of money on collecting data and extra labor to annotate the target objects in the image. The mixed dataset will be augmented to increase the amount of data in the limited dataset by the method of data augmentation, such as flip, contrast, and brightness adjustment. Furthermore, this paper utilizes Inverse Perspective Mapping (IPM) to produce bird’s eye view images of the scene from the front-view image plane. Compared to the front-view images for testing, the transformed images eliminate the perspective distortion so that the accuracy of object detection can be improved.

The contributions of the paper are listed below:

1. A virtual dataset mixed with an open dataset is viable for cross-field detection when training them together and testing them on the real dataset.
2. The method of data augmentation is employed to increase the amount of data in the limited dataset without extra labor to annotate the target objects in the image.
3. The integration of Inverse Perspective Mapping (IPM) to transform input images into a bird’s eye view is a key innovation of this work, significantly improving road marking detection accuracy. This approach addresses perspective distortion, resulting in a remarkable mAP improvement from 60.04% to 78.66%.
4. The Mask R-CNN tests the front-view images and the bird’s eye view images, and the mAP increases from 60.04% to 78.66%, which is a significant improvement in model accuracy.

The following section is the organization of the paper. In Section 2, literature reviews of object detection algorithms and road markings detection are introduced. In Section 3, the methodology of the proposed approach is elaborated in detail. Section 4 evaluates the proposed method by the experiments and analyzes the results. In Section 5, conclusions and future works of the paper will be mentioned.

## 2. Related Work

Some previous research works are reviewed in this section, which consists of two major sections: object detection algorithms and research on road markings detection. The following will elaborate on the development of the object detection algorithm, including the one-stage model and two-stage model. In addition, some existing research on road markings will also briefly be reviewed.

### 2.1. Object Detection Algorithms

Convolutional Neural Network (CNN) algorithms are extensively applied in tasks such as object recognition, classification, semantic segmentation, etc. Object detection is the task of identifying an object with a certain class and localizing the object’s position in the image. There are two major categories of object detection models: one-stage models and two-stage models. One-stage models emphasize speed performance, e.g., YOLO [1]. Two-stage models emphasize detection accuracy, e.g., R-CNN [2], Faster R-CNN [3], and Mask R-CNN [4]. The one-stage and two-stage models are described in detail below.

#### 2.1.1. Two-Stage Model

CNN-based image recognition finds applications across various domains. Extracting details about an object’s location and size within the image is crucial. The sliding window technique, a straightforward approach, involves scanning the image with a fixed-sized box and employing CNNs for object identification. In order to obtain better results, it is necessary to use different sizes of the box; however, this process is extremely slow. Accordingly, an increasing number of methods were proposed. R-CNN, proposed by Ross Girshick et al., generates about 2000 candidate Region Proposals for the whole image, and each candidate region is fed into the CNN for feature extraction. Since it takes a great deal of time to retrieve features from more than 2000 candidate regions that are overlapped, Ross Girshick modified the network by adding ROI pooling, calling it Fast R-CNN [5], which calculates the feature values of the whole image at once and corresponds to the actual location of the candidate regions to obtain the feature values of each region. The training process is simplified and saves quite a lot of computing time. Due to the fact that generating multiple candidate regions is quite time-consuming, Shaoqing Ren et al. presented Faster R-CNN to shorten the overall computation time. Faster R-CNN takes advantage of the Region Proposal Network (RPN) to generate the candidate regions effectively. The anchor box and probability of the box will be the output to classify each candidate region, which corresponds to the actual location of the candidate region. Previous approaches aim to frame the object, while Mask R-CNN, proposed by Kaiming He et al., can produce pixel-level masks. In the research, Mask R-CNN is implemented to train an object detection model. The detailed information will be introduced in the methodology.

#### 2.1.2. One-Stage Model

In the above evolution of the two-stage model, the overall processing tasks also achieve the ability to generate pixel-level masks with a decent speed. Joseph Redmon et al. proposed YOLO (You Only Look Once), focusing on prediction speed rather than accurate masks. YOLO is designed for end-to-end training. Not only does it make training easier but also faster. With the rapid development of YOLO architecture, YOLOv1, v2 [6], v3 [7], and v4 [8] have been released. YOLOv2 introduced several enhancements over YOLOv1, including improved accuracy, faster processing, and the ability to detect a greater variety of objects. In 2018, Joseph Redmon et al. further advanced the model with the release of YOLOv3. YOLOv3 introduced Residual Network (ResNet) [9] and Feature Pyramid Network (FPN) [10] to solve the gradient vanish problem and to optimize small object detection by combining different scales of feature maps resulting in more accurate results than the previous versions. YOLOv4 was released by Alexey Bochkovskiy et al., enhancing various parts of YOLOv3. In addition to maintaining the speed, the detection accuracy was significantly strengthened.

Mask R-CNN, which is improved by Faster R-CNN, burnishes its legacy on the instance segmentation model. Influenced by Mask R-CNN, Daniel Bolya et al. hoped to design a one-stage instance segmentation model that integrated the merits of Mask R-CNN and YOLO, namely YOLACT [11]. YOLACT is a real-time instance segmentation model, which performs the object detection tasks optimally and computing speed rapidly. YOLACT splits the complex instance segmentation process into two simple parallel tasks, generating prototype masks and predicting mask coefficients per instance. For each instance, the corresponding predicted mask coefficient is simply multiplied and added to the prototype mask. Subsequently, the instances are filtered according to the bounding box and the threshold value to obtain the corresponding mask for each instance, which is a high-quality mask. SOLO [12] directly segments the instance mask, which is a box-free approach. SOLO considers a method that introduces the concept of instance categories to predict the class of object instances. To distinguish the object instances based on their center locations and object sizes, the approach transforms the instance segmentation issue into a classification issue, imitating the idea of semantic segmentation to predict the class of each pixel. The accuracy of the experiment testing on the COCO dataset has surpassed Mask R-CNN. SOLO version 2 [13], published by the same author, improves the mask learning and NMS (Non-Maximum Suppression) approach, which not only enhances the accuracy but also realizes the real-time requirements.

### 2.2. Research on Road Markings Detection

Road markings detection has been the popular research in the field of autonomous driving for decades. Most of the previous research has concentrated on the detection of lane lines instead of the road markings, such as pedestrian and road speed limit markings. In this section, handcrafted and deep-learning methods of road markings works will be introduced first. Next, road markings detection based on Inverse Perspective Mapping will be explained in the following part.

#### 2.2.1. Research on Road Markings Detection Based on Handcrafted and Deep-Learning Methods

The prior research on road markings detection is roughly classified into two categories, one is handcrafted features methods, and the other is deep-learning-based object detection methods. Conventional methods for road markings detection tasks mostly extract the basic feature of the target object manually, e.g., color, edge, and texture, which vastly rely on the method that the author designed. For instance, Tang et al. [14] utilized a Histogram of Oriented Gradient (HOG) [15] and Support Vector Machine (SVM) [16] with the Region Of Interest (ROI) restrictions, which demonstrated good performance on the dataset. In contrast to the handcrafted methods, the deep-learning-based approach indicates better results and stability in the feature extraction of road markings. Object detection based on CNN has apparently improved the performance under various situations. VPGNet [17] is an end-to-end model that detects vanishing points and road markings on the road surface. Furthermore, the author released a new dataset that is publicly available collecting data under various weather conditions in Korea. Hoang et al. [18] detected and classified the arrows and bike markings on the road based on the adaptive ROI and RetinaNet. The results show that the adaptive ROI outperforms other methods. To pursue real-time detection, Zhang et al. [19] proposed a method consisting of three modules: preprocessing, road markings detection, and segmentation. A lightweight network combined with the Siamese Attention module is adopted to improve the accuracy and enhance the sensitivity to road markings in the second stage. For the segmentation module, the segmented objects can reach pixel-level accuracy and cost less computation. Ye et al. [20] proposed a two-stage model, YOLOv2 combined with a Spatial Transformer Network (STN) [21], to tackle the distortion of road markings. The presented method can obtain good performance with less computation even though it is a two-stage model. In summary, the deep-learning-based approach is more robust and more stable than the traditional feature extraction approach and can be applied to different scenarios with higher accuracy.

#### 2.2.2. Road Markings Detection Based on Inverse Perspective Mapping

In order to drive the car automatically, autonomous cars must be able to perceive the surrounding environment at any time and handle emergency conditions. In existing methods, detection accuracy usually decreases with increasing distance; thus, objects are relatively smaller. Moreover, scales of elements on the road are inconsistent at different distances and perspectives, while distorted elements occur in the distance. The perspective distortion can be eliminated by Inverse Perspective Mapping (IPM), which transforms the image into bird’s eye view (BEV) and can solve the problem stated above. The existing research on road marking detection often adopts IPM to obtain BEV images during the preprocessing stage so as to reduce the complexity of the original image and remove the unwanted parts of the image that focus on the ROI. Li et al. [22] performed the IPM transformation to eliminate the impact of the perspective effect. ROIs extracted from IPM images are exploited to detect the road markings. Greenhalgh et al. [23] presented a method that can detect the road markings text and symbols automatically. Before detecting the targets, the images were transformed into an IPM image to remove the perspective distortion. MSER (Maximally Stable Extremal Regions) [24] could subsequently be applied to generate candidate regions. Symbol-based road markings were recognized by HOG and SVM, while text-based road signs were identified by the optical character recognition (OCR) package. Bailo et al. [25] applied the MSER to obtain candidate regions under different illumination and weather conditions. The proposed method is proven to detect the object on the image robustly. Kang et al. [26] considered a method to reach the real-time detection of road markings based on the YOLOv2 model. The synthetic dataset constructed by the MSER algorithm contains classes and orientations, trained by the detector to predict the class labels and position. A review of the common characteristic of IPM-related references reveals the transformation of the front-view image to a bird’s eye view. The purpose of the transformation to a bird’s eye view is to extract the ROI more easily and then perform manual feature extraction. In addition, the current literature uses front-view images to train deep-learning models and converts front-view images to top-view images without considering the problem of disparity in viewing angles. Therefore, this study considers the relationship between different view angles and normalizes the view angle to the top view, which significantly improves the model performance and helps to improve the object detection accuracy.

## 3. Methodology

**Figure 1:**
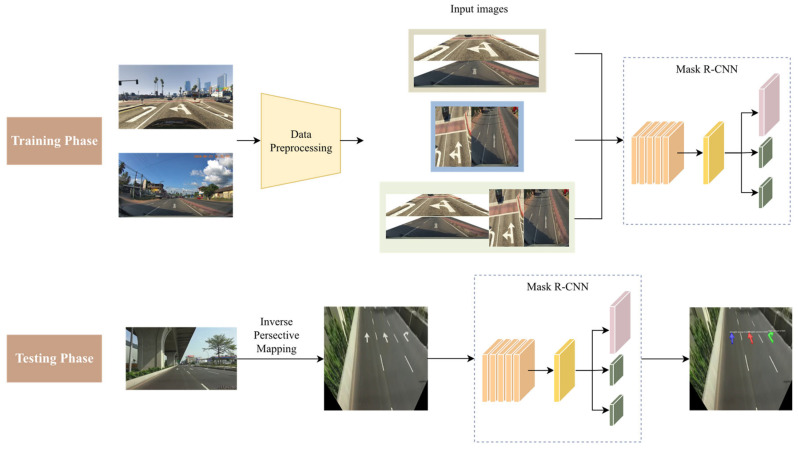
Illustration of the proposed method.

In this section, the overall proposed method will be introduced first. Figure 1 provides an apparent visualization of the overall framework of the proposed method. The procedure is divided into two phases, the training phase and the testing phase. For the sake of the inconvenience of collecting data, the training data are composed of the two open datasets for cross-field detection. Additionally, in order to focus on the objects of the road surface, the mixed dataset will be processed in the training phase. The images will be cropped to remove the irrelevant background, and afterward the Inverse Perspective Mapping (IPM) will be applied to project to the perspective of the bird’s eye view, which is favorable for the object detection model. For the testing phase, IPM will be performed on the testing data, projecting the images to the bird’s eye view.

### 3.1. The Proposed Method

**Figure 2:**
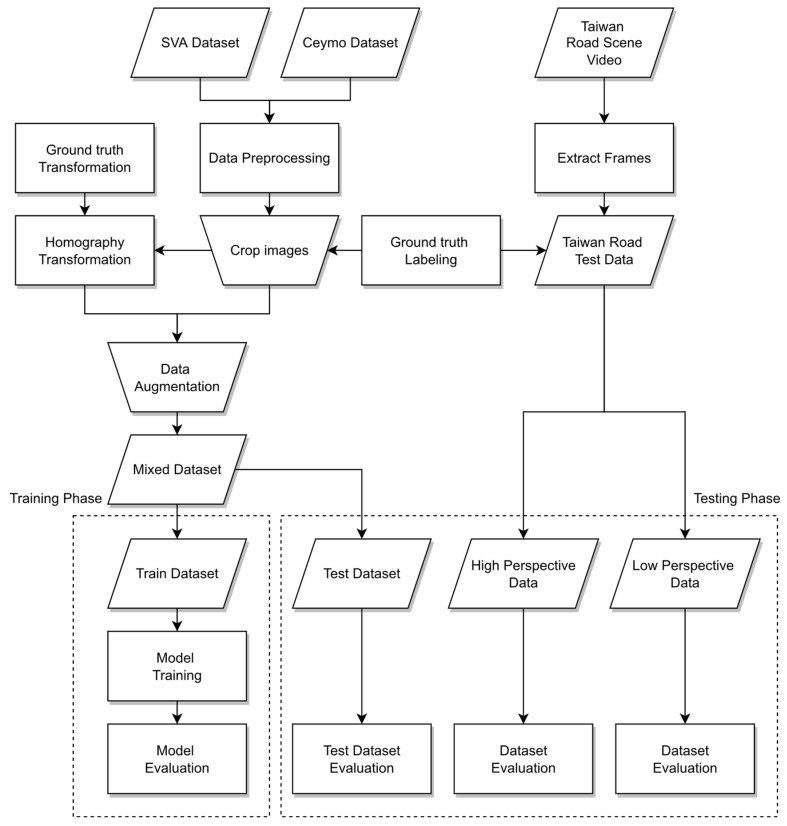
The overall pipeline of the proposed method.

The pipeline of the proposed method will be manifested in this chapter. Figure 2 illustrates the workflow of the research, including input, output, and method. Three datasets are utilized in the research including the Surrounding Vehicles Awareness (SVA) dataset, the Ceymo dataset [27], and the Taiwan road scene data. The SVA and Ceymo datasets will be mixed into one dataset for the training phase, while the Taiwan road scene dataset extracted from the YouTube video are testing data. Before training the model, two of these datasets will be preprocessed comprising data augmentation, homography transformation, and ground-truth labeling. After data preprocessing, the mixed data will be fed into the segmentation model for training and testing on the real dataset. The proposed method can be implemented in any of the instance segmentation models and even in semantic segmentation models to reach the demand of pixel-level output. Three different types of models will be trained in the instance segmentation model and compared with each other. The first one is the front-view model in which most of the previous studies primarily use front-view images as input. The second one trains the bird’s eye view images as input, which are transformed by the front-view images. Unlike previous studies, this paper uses images from different viewing angles as training data. The third one takes the front-view images and bird’s eye view images as input such that the model can detect the different perspectives of images that make the model more robust, general, and stable. In terms of user convenience, they may not necessarily convert the images to top-view, so this paper also proposes a third method to balance the cross-field detection at different perspectives. For the testing phase, in order to solve the issue of missed recognition caused by different perspectives and far distances, before testing the Taiwan road data, the image will be transformed into the perspective of the top-view. Thus, the distorted objects can be presented in their entirety to improve the accuracy of the prediction.

### 3.2. Homography Transformation Based on Inverse Perspective Mapping

**Figure 3:**
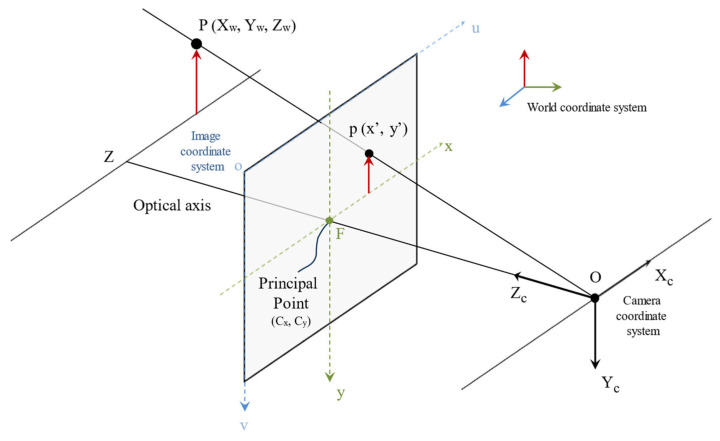
Pinhole camera model.

In object detection tasks based on the deep-learning method, the problem of “small objects at far distance” is hard to solve. Therefore, IPM is applied to remove the perspective effect and reduce the disparity between the front-view and bird’s eye view. IPM relies on two assumptions: (1) the camera is in a fixed position relative to the road, and (2) the road surface is flat. The main concept of IPM is to project the world coordinate system to the 2D (two-dimensional) coordinate system. The pinhole camera model (Figure 3) plays a crucial role in the concept of perspective transformation. In the pinhole camera model, parameters are described as follows, where ${P(X}_{w},Y_{w},Z_{w})$ is an object of the world coordinate system, and $p(x^{\prime },\;y^{\prime })$ is a 2D point on the image coordinate system when P is being projected onto the image. The distance between F and O is called the focal length, where O represents the center of the camera, and F represents the center of the image. It describes the coordinates of a real-world object transform as stated in the following: the coordinates of the object in the 3D (three-dimensional) world are transformed to the coordinates of the camera first, and then transformed to the coordinates of the 2D images on the 2D plane. In brief, the pinhole camera model depicts how an ideal pinhole camera with an infinitely small aperture projects an object in the 3D world onto a 2D plane.

The transformation matrix is formulated by the camera parameters, intrinsic parameter matrix, and extrinsic parameter matrix. For the intrinsic parameter matrix *K* (1), $f_{x}$ and $f_{y}$ are the focal lengths of the camera, and the $c_{x}$, $c_{y}\;$ are the optical centers of the camera. It describes the geometric description of light inside the camera and converts the camera coordinates to the image coordinates. First, it scales the image plane from a unit of object space coordinates into pixels. Second, it shifts the original point from the middle of the image into the top-left corner.

(1) $$K=\left[\begin{array}{ccc} f_{x} & 0 & c_{x}\\ 0 & f_{y} & c_{y}\\ 0 & 0 & 1 \end{array}\right]$$

The extrinsic matrix $C_{ext}$ (2) is composed of rotation matrix R and translation matrix *T*, which describe the orientation and location of the camera in the world system. In brief, it indicates the transformation from world coordinates to camera coordinates.

(2) $$C_{ext}=\left[\begin{array}{c} R\left|T\right. \end{array}\right]=\left[\begin{array}{cccc} r_{11} & r_{12} & r_{13} & t_{x}\\ r_{21} & r_{22} & r_{23} & t_{y}\\ r_{31} & r_{32} & r_{33} & t_{z}\\ 0 & 0 & 0 & 1 \end{array}\right]$$

The points in the world are (*X*, *Y*, *Z*) coordinates that are turned into homogeneous forms. After multiplying by two matrices, the homogeneous coordinates of the pixel in the image are calculated. Hence, combining two matrices by matrix multiplication is the camera matrix shown in (3), which is a 3 × 4 matrix with 12 numbers, denoted as $C_{ij}$. The elements $C_{ij}$ represent the intrinsic and extrinsic parameters of the camera, where i is the row index and j is the column index. λ is an arbitrary scalar scale factor that scales the ($x^{\prime },y^{\prime },z^{\prime }$) coordinates. The ($x^{\prime },y^{\prime }$) are divided by $z^{\prime }$; thereforethe λ disappears. The coordinates multiplied by the matrix use any arbitrary scale factor and will obtain exactly the same result. Since the scale factor is arbitrary, the element in *C* (3, 4) will be set to 1.

(3) $$\left[\begin{array}{c} x^{\prime }\\ y^{\prime }\\ z^{\prime } \end{array}\right]=\lambda \left[\begin{array}{cccc} C_{11} & C_{12} & C_{13} & C_{14}\\ C_{21} & C_{22} & C_{23} & C_{24}\\ C_{31} & C_{32} & C_{33} & C_{34} \end{array}\right]\left[\begin{array}{c} X_{w}\\ Y_{w}\\ Z_{w}\\ 1 \end{array}\right]$$

The matrix (4) can be simplified to consider the camera projection on a point on a plane of the world space; therefore, the *Z* value of the points will be set to 0 and the third column of the matrix will be multiplied to be 0. After removing a row of the vector, it is a 3 × 3 system called planar Homography H. It maps the coordinates of the points in a plane to the points in the image. Two-dimensional coordinates of a point in the world can be converted by using the simple matrix into the coordinates of a point in the image.

(4) $$\left[\begin{array}{c} x^{\prime }\\ y^{\prime }\\ 1 \end{array}\right]=\left[\begin{array}{ccc} C_{11} & C_{12} & C_{14}\\ C_{21} & C_{22} & C_{24}\\ C_{31} & C_{32} & 1 \end{array}\right]\left[\begin{array}{c} X_{w}\\ Y_{w}\\ 1 \end{array}\right]=\left[\begin{array}{ccc} h_{11} & h_{12} & h_{13}\\ h_{21} & h_{22} & h_{23}\\ h_{31} & h_{32} & 1 \end{array}\right]\left[\begin{array}{c} X_{w}\\ Y_{w}\\ 1 \end{array}\right]$$

Homography transformation is described by the following equations, where *H* is the 3 × 3 matrix with eight degrees of freedom (DoF), so the H can be estimated from at least four world points and their corresponding image points. Among these four points, any three of them should not be collinear. $x^{\prime }\;{(x}^{\prime },\;y^{\prime })$ represents transformed coordinates, and $\tilde{x}{(X}_{w},Y_{w},1)$ represents homogeneous coordinates. The Homography matrix can warp image pixel coordinates to corresponding pixel coordinates on the target image. The target coordinates are calculated by the collinear equations using least-squares estimation.

(5) $$x^{\prime }=H\tilde{x}\;H=K\left[\begin{array}{c} R\left|T\right. \end{array}\right]\;H=\left[\begin{array}{ccc} f_{x} & 0 & c_{x}\\ 0 & f_{y} & c_{y}\\ 0 & 0 & 1 \end{array}\right]\left[\begin{array}{ccc} r_{11} & r_{12} & t_{x}\\ r_{21} & r_{22} & t_{y}\\ r_{31} & r_{32} & t_{z} \end{array}\right]=\left[\begin{array}{ccc} h_{11} & h_{12} & h_{13}\\ h_{21} & h_{22} & h_{23}\\ h_{31} & h_{32} & h_{33} \end{array}\right]$$

The eight equations are used to estimate the eight degrees of freedom in the homography matrix, while $h_{33}$ is set to 1. The $h_{ij}$ is shifted to another reference frame, and the coordinates of the 2D image are mapped according to (5), which will be calculated by the least-squares method. After applying homography transformation to the front-view images, the images will be projected onto the bird’s eye view. The four yellow points are decided along the roadside, so the ROI will focus on the road area.

In the research, the homography transformation is performed on the data augmentation to train the model. For the testing phase, the testing data will also operate the homography matrix to transform the different angles to the perspective of the bird’s eye view. In conclusion, IPM transforms 2D images into other 2D images on the same planar surface through homography matrix multiplications so that the images from the monocular camera as the front-facing image will be projected onto top-view images.

### 3.3. Data Augmentation

Data augmentation is a common method that is used in the deep-learning model for the purpose of increasing the number of images from small datasets to avoid overfitting the specific scene. To begin, the data augmentation on images will be explained. Owing to the change of pixel coordinates, the data augmentation on the label will also be expounded in the next part.

#### 3.3.1. Data Augmentation on Images

Deep-learning networks generally require a great deal of training data to obtain better results. With the limitation of obtaining data, data augmentation is utilized to produce more data from existing datasets so that the diversity of the original images is increased to make up for the lack of data. Common techniques of data augmentation include (1) geometric transformation: randomly flip, crop, rotate, shear or translate images; (2) color space transformation: change the color channel space or try to map the RGB to other color spaces; (3) noise injection: a matrix of random values sampled from the Gaussian distribution is added to the RGB pixels of the image; and (4) kernel filters: kernel filters on images for convolution operations, such as sharpen and blur. After data augmentation, people looking with their eyes still easily recognize the same image; however, for the deep-learning model, these processed images are completely new images.

Considering the simple features and monotonous color of the road markings, they do not contain diverse structural features for the object detection model. Accordingly, this paper adopts the flip and color space adjustment (brightness and contrast) to the training dataset. Flip is one of the effective methods and has proven to be useful to improve the performance of the deep-learning model. Furthermore, color space adjustment is the easiest and most common technique to change the luminance of images. On the road surface environment, the diversity of lighting conditions and weather conditions have an impact on the accuracy of the model, and hence, data augmentation is a vital technique to vary the images by the color space adjustment. The images are flipped using horizontal flipping and vertical flipping, which are common methods in geometric transformation. Moreover, brightness adjustment is implemented to the training data to convert the brightness-related channel depending on the value setting. Therefore, it can make the images slightly brighter or darker to enhance the lighting conditions of images. Contrast adjustment is also one of the data augmentation techniques that rescales the range of intensity value in the images. The contrast is the ratio between the brightest and darkest areas of the images. The lager the ratio, the more gradations from black to white, which makes the object or the boundary in the images more distinguishable. Therefore, the contrast of the white road markings on the black asphalt road should be enhanced, which will improve the visual perception. Ultimately, four sets of image copies were generated by the data augmentation techniques, which increased the amount of data from the original images without extra time costs.

#### 3.3.2. Data Augmentation on Label

Labeling the ground truth of target objects is necessary for the supervised learning network. Before training the model, target objects of the dataset need to be labeled as ground truth. Nevertheless, deriving more images is difficult and they also need to be labeled. It is time-consuming and requires a considerable amount of commitment. As a consequence, the study increases the amount of data using data augmentation and the homography transformation of images in the limited dataset. The annotations do not require additional manual labeling after data augmentation and homography transformation. In view of the pixel coordinates change, some labels of the augmented data need to be modified. After data augmentation such as brightness and contrast adjustment, the label is the same as the original annotation files, whereas the pixel coordinates of the flip images need to be horizontally flipped. If the label originally corresponds to a “right arrow”, it will be changed to a “left arrow” after the flip. The pixel coordinate of flipped images is converted from the left part to the right part. Furthermore, the original images from the mixed dataset will be transformed into the perspective of the bird’s eye view. Consequently, the pixel coordinates of the bird’s eye view label are transformed from the front-view label using the inverse of the homography matrix.

### 3.4. The Architecture of Mask R-CNN for Road Markings Detection

**Figure 4:**
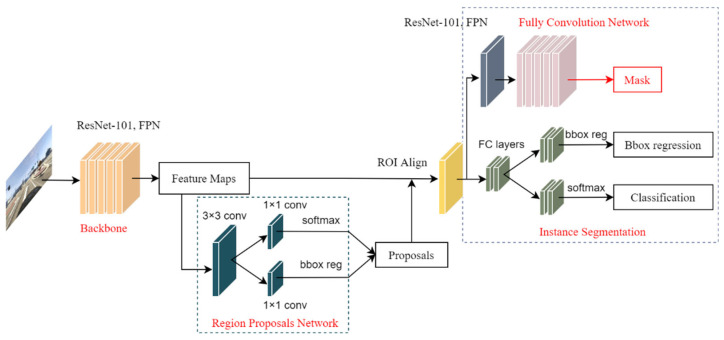
The architecture of Mask R-CNN.

Mask R-CNN is a classic instance segmentation model. The merit of the Mask R-CNN is pursuing the prediction accuracy, which can reach pixel-level accuracy. Compared to other semantic models, Mask R-CNN is more robust and adaptive to different datasets. The one-stage models mainly pursue the real-time requirements but often achieve bad prediction results. Compared to the one-stage models, the region-base of two-stage models focuses on prediction accuracy. In light of achieving a better result, Mask R-CNN is noted as an influential instance segmentation model that can be used to detect the road markings on the road surface. Mask R-CNN has roughly the same framework as Faster R-CNN. The difference between Faster R-CNN and Mask R-CNN is that the original two tasks are changed to three tasks: classification, regression, and segmentation. ROI pooling is replaced by the ROI align. ResNet with 101 layers in combination with the neck of the Feature Pyramid Network (FPN) is used as the feature extraction backbone in the implementation. FPN is a top-down architecture with skip connection that solves problems at different scales, which is beneficial to small object detection. Mask R-CNN is divided into two stages: finding the region proposals first and then identifying them. The first stage has the same first layer as Faster R-CNN, called Region Proposal Network (RPN). RPN consists of two branches. One of the branches determines the probability of whether the anchor contains an object or not. The other branch is responsible for calculating the offsets (x, y,w,h) between the anchors and ground truth. The output of RPN will be the input into the ROI align. A modification is proposed to improve the location accuracy known as ROI align. Using bilinear interpolation of the virtual pixel at the point with the nearest pixel—instead of using the quantization output for obtaining a fixed size from ROI pooling—solves the problem of misalignment, which is caused by twice quantization in the ROI pooling operation. Therefore, the accuracy of the bounding box location makes obvious progress. In the second stage, apart from predicting classes and bounding box locations, a branch of the fully convolutional network is added. A corresponding binary mask is predicted for each ROI to indicate whether a given pixel is part of the target or not. The overall architecture of Mask R-CNN is depicted in Figure 4.

The loss of Mask R-CNN consists of three losses (6), including classification loss, bounding box loss, and mask loss. The classification loss and bounding box loss originates from Faster R-CNN. The loss of Faster R-CNN defined in (7) is divided into two parts, classification loss and regression loss. Classification loss is calculated as the average binary cross-entropy, comparing each predicted probability ($P_{i}$) to the actual class output, which is either 0 or 1. For regression loss, it is the value of the bounding box offset, where $t_{i}=\left\{t_{x},t_{y},t_{w},t_{h}\right\}$ are four coordinates of the predicted bounding box while $t_{i}^{*}$ is the ground-truth box linked to a positive anchor. It adopts SmoothL1 loss to train the model. The mask loss of Mask R-CNN allows every ROI to generate all the masks for every class; however, not every mask output will contribute to the loss. Instead, the mask loss of class k will be counted according to the prediction result of the classification branch in class k. The perspective transformation is not limited to using Mask R-CNN as the training model. Given the maturity and flexibility of the Mask R-CNN, as well as the characteristics of basic features and the monotonous color of the road markings, it is suitable for deployment.

(6) $$L=L_{cls}+L_{box}+L_{mask}$$

(7) $$L(\left\{p_{i}\},\{t_{i}\right\})=\frac{1}{N_{cls}}\sum_{i}L_{cls}(p_{i},p_{i}^{*})+\lambda \frac{1}{N_{reg}}\sum_{i}p_{i}^{*}L_{reg}(t_{i},t_{i}^{*})$$

## 4. Experimental Evaluations

In the first place, the experimental settings and evaluation metrics for the proposed method will be described. Secondly, this section elucidates the detailed information of the datasets in the experiment. Finally, some experiments and results of the proposed method are presented at the end of the section.

### 4.1. Experimental Settings

The study is implemented with the Linux Ubuntu 18.04 platform using the Tensorflow-gpu-1.12 deep-learning framework, and an NVIDIA GeForce RTX 2080-Ti graphics card unit (GPU). The hardware specifications are displayed in Table 1.

The following is an empirical setup for the model’s hype-parameters: the number of steps per epoch is the total training samples divided by the batch size, where the total epochs are 100, and the learning rate is 0.001. Other training settings are shown in Table 2.

To evaluate the performance of the proposed method, the widely used evaluation metric Mean Average Precision (*mAP*) was employed to evaluate the model. The *mAP* is related to the four metrics of *IoU*, *Precision*, *Recall*, and *AP*. The following describes each metric and its formula:

1. *IoU* (Intersection over Union): Equation (8) displays the overlap ratio between the ground-truth bounding boxes and predicted bounding boxes. The higher the overlap ratio, the higher the accuracy of the predicted target object position. Essentially, the predefined threshold is 0.5.

(8) $$IoU=\frac{Area\;of\;Overlap\;}{Area\;of\;Union\;}$$

2. *Precision*: *Precision* (9) is the number of predicted objects that have been predicted as positive where true positive (*TP*) is the predicted object that matches the ground-truth objects, and false positive (*FP*) is the positively predicted object that is actually false.

(9) $$Precision=\frac{TP}{TP+FP}$$

3. *Recall*: *Recall* (10) is the number of actual objects that the model predicts correctly, where false negative (*FN*) represents when the model predicts a negative object that is actually positive.

(10) $$Recall=\frac{TP}{TP+FN}$$

4. *AP* (Average Precision) represents the area under the *precision*–*recall* curve (PR curve). According to PASCAL VOC [28] competitions after 2010, the calculation of *AP* (11) has a modification that selects the maximum *precision* value at unique *recall* values. In this case, the *AP* is computed by interpolating the *precision* across all points n, and r takes the maximum *precision* when the *recall* value is greater than or equal to *r* + 1, as shown in (12); $\rho (\tilde{r})$ is the measured *precision* at *recall* $\left(\tilde{r}\right)$.

(11) $$AP=\sum_{n=0}\left(r_{n+1}-r_{n}\right)\rho_{interp}(r_{n+1})$$

(12) $$\rho_{interp}\left(r_{n+1}\right)=\underset{\tilde{r}:\tilde{r}\geq r_{n+1}}{\max}\rho (\tilde{r})$$

5. *mAP* (Mean Average Precision): the average of the *AP* for every class. The *mAP* as shown in (13) is a principal quantitative measurement for object detection.

(13) $$mAP=\frac{1}{N}\sum_{i=1}^{N}AP_{i}$$

### 4.2. Dataset

**Figure 5:**
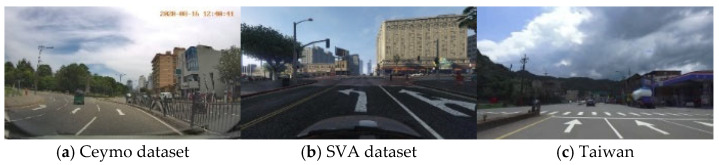
Example of images from different datasets.

In this section, two datasets (Ceymo and SVA datasets) for training and the Taiwan dataset for testing will be introduced. Two open datasets were collected in Sri Lanka and in the virtual world, Grand Theft Auto V (GTAV), respectively. The Ceymo dataset consists of 2099 images for training and 788 for testing belonging to eleven classes, providing polygon, bounding box annotations, and pixel-level segmentation masks. In order to satisfy our research demand, we only retained seven classes and selected 2172 images with annotations. Additionally, the Surrounding Vehicles Awareness (SVA) dataset was collected from the virtual world, GTAV, simulating real-world scenarios under abundant weather conditions and different illuminations. We selected 1771 images from the SVA dataset and labeled them into six classes. For the SVA dataset, the whole labeling procedure was performed manually by the image annotation tool, VGG Image Annotator (VIA). As for the Ceymo dataset, it provides label files annotated by Labelme, which is a different format from VIA; therefore, the label files from the Ceymo dataset need to be transformed into the format of VIA in view of uniformity. The total images within the two datasets are 3943. Figure 5a,b depict some examples from each dataset. Subsequently, the datasets were mixed into one dataset and were randomly divided into training sets and validation sets of each dataset with a proportion of 7:3, containing, in total, 2785 training data and 1158 validation data, respectively.

**Figure 6:**
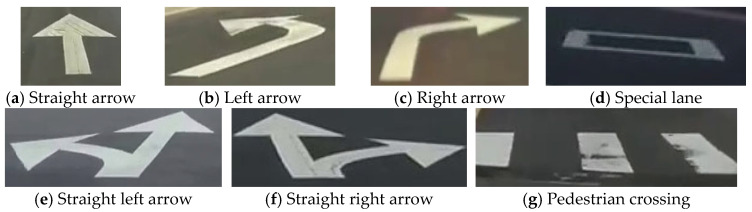
Classes of Taiwan road images (seven classes).

The testing data were derived from YouTube videos in the field of the Taiwan road scene. Figure 5c shows some examples of images from the Taiwan dataset. The data contain diverse scenarios at different times during the days, including sunny, rainy, and cloudy. The total images in the testing data consist of 582 images. Figure 6 presents the seven classes in the Taiwan data for model prediction, including straight arrow, left arrow, right arrow, straight left arrow, straight right arrow, special lane, and pedestrian crossing, which were labeled manually via the VIA tool.

### 4.3. Data Preprocessing

Before training the model and testing the images, the images and label files will be preprocessed first. Four approaches of data augmentation will augment the total quantity of images. In addition, the homography transformation based on Inverse Perspective Mapping (IPM) is applied to the training data and testing data.

#### 4.3.1. Data Augmentation

**Figure 7:**
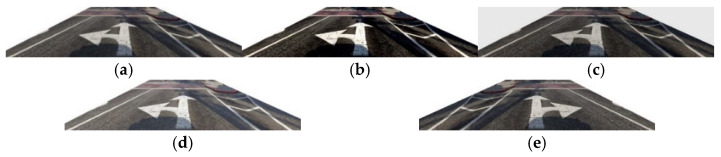
Data augmentation. (**a**) Original image. (**b**) Contrast. (**c**) Dark. (**d**) Bright. (**e**) Flip.

The experiment used the data augmentation method only on the training set, and the testing set consists of the original images. Data augmentation was performed by changing the contrast and brightness of the images in the experiment. The experiment was realized by the Imgaug package. The Imgaug package is a python library for image augmentation providing the keypoint and bounding box transformation. There are three functions adopted in the experiment, inclusive of “AddToBrightness” function, “LinearContrast” function, and “Fliplr” function. The LinearContrast function sets the alpha value to sample uniformly within the specific interval [0.4, 1.6]. In the experiment, we set 1.6 as the contrast value to adjust the intensity of images. Figure 7b illustrates the outcome after contrast adjustment. The AddToBrightness function converts each image to a color space with a brightness-related channel, extracts the channel, and then adds or subtracts the channel value between −30 and 20 to convert it back to the original color space. Figure 7c illustrates the dark image reducing the lighting, and Figure 7d illustrates the bright image. The flip function can flip the images horizontally or vertically. Fliplr means to reverse the images from left to right, which horizontally flipped the images. Figure 7e shows the example of the horizontally flipped image. Data augmentation is helpful to increase the amount of data in the limited dataset without extra labor to annotate the target objects in the image. After data augmentation, the number of images increased from 2785 to 13,925.

#### 4.3.2. Inverse Perspective Mapping

**Figure 8:**
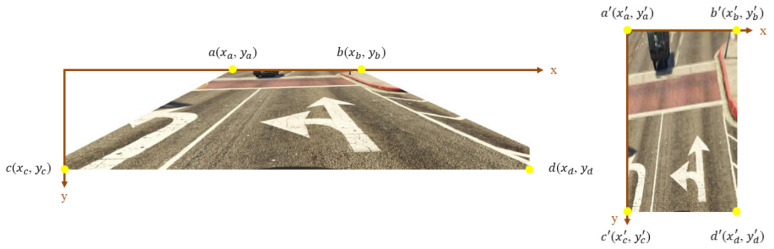
Source points $\left(x_{i},\;y_{i}\right)$ and reference points $\left(x_{i}^{\prime },\;y_{i}^{\prime }\right)$ within the different perspectives of the images.

The homography transformation based on IPM is employed to the Ceymo dataset and SVA dataset to transform the images into the bird’s eye view to augment the dataset. The two datasets do not provide the camera parameters, so the perspective transformation adopts planar projective transformation, choosing four points on the input image and the corresponding points on the output image to estimate the homography matrix. The input image size affects the learning of the object detection model. Compared to small images, large images not only require more training time and more memory to extract the internal features of the image but also contain more background noise, which has a negative impact on detection. Consequently, the images will be cropped first in order to remove the irrelevant background so that the model can focus on the road surface, which reduces the effect of the environmental conditions and unnecessary feature learning. Furthermore, the reserved ROI easily chooses the source point to map to a corresponding point in the target image through perspective transformation. OpenCV provides perspective transformation functions to calculate the homography matrix for the images given the source and destination points. The “getPerspectiveTransform” function computes the projection matrix. Afterward, the top-view perspective transformation is performed using the “warpPerspective” function. Figure 8 provides the example of source points $\left(x_{i},\;y_{i}\right)$ in the cropped image and reference points $\left(x_{i}^{\prime },\;y_{i}^{\prime }\right)$ in the bird’s eye view image. The pixel coordinates of the points are transferred from one plane to another through homography matrix multiplication.

**Figure 9:**
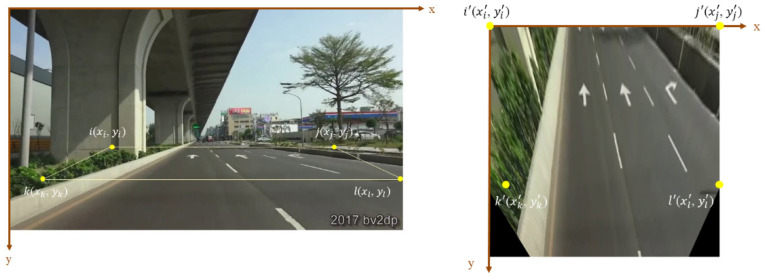
Source points $\left(x_{i},\;y_{i}\right)$ and reference points $\left(x_{i}^{\prime },\;y_{i}^{\prime }\right)$ in the Taiwan dataset.

As for the testing phase, the proposed method also transforms the data into the bird’s eye view using IPM. Figure 9 schematizes the location of source points $\left(x_{i},\;y_{i}\right)$ and destination points $\left(x_{i}^{\prime },\;y_{i}^{\prime }\right)$. The four yellow points are decided along the roadside so that the ROI will focus on the road area. To ensure the fairness of the experiment, the number of the label’s samples after transformation is the same.

### 4.4. Experimental Results

This section describes the experimental results, including a quantitative analysis of the proposed method, in comparison with other models, and examples of the detection results. For training on an NVIDIA GeForce RTX2080 Ti with a dataset of 2785 training images and 1158 validation images, the training time for 100 epochs is approximately 1 h 20 min. The inference time for the validation set is about 1–2 min, with each image taking approximately 70–120 ms to process.

#### 4.4.1. The Cross-Field Detection

The evaluation of the proposed method for cross-field detection is measured in this section. As explained in the 4.2 dataset, all images for training data that are derived from two open datasets were mixed in one dataset. Table 3 and Table 4 display the comparison of same-field and cross-field detection results. In the experiment, the research trained three kinds of images as training data. The first one (Table 3) was the front-view model that primarily used front-view images as input in previous methods. Testing in the same field, the mAP was 98.22%. For the cross-field detection, testing on the Taiwan road surface, the model had the detection ability for the mAP to reach 57.90%. Different datasets had different angles of the camera, so the data tested in different fields would have deviations in angle view, resulting in poor detection. Therefore, the second one (Table 4) fed the bird’s eye view images into the deep-learning model occupying an important place. Testing in the same-field data, the model performance was 97.02%, while testing in the Taiwan data, the model performance was 85.57%, which is a good detection capability. The mAP of the model was improved by 27.67% compared to the detection ability of the first model with the same viewing angle. This also proves that after normalizing the perspective into a bird’s eye view, the results significantly improved even when tested on different datasets. Although the mAP of the cross-field detection is not as high as the same-field detection, the model has the detection ability to be utilized in the different field. Using open data from different fields not only saves time in data collection but also proves the feasibility of cross-field detection.

Despite the fact that the model has the ability to detect the object on the Taiwan road, the model still has room for improvement. Due to the different perspectives and the small objects at a far distance, some objects have difficulty being predicted; thus, IPM is a vital method to improve this situation. The experimental results will be presented in the forthcoming sections.

#### 4.4.2. The Comparative Results of Different Perspectives

Table 5 shows the mean average precision (mAP) of the proposed method and the different models using the proposed method. The difference of accuracy after perspective transformation testing on the Taiwan data will be listed in the table. First of all, the mAP of the front-view images is 57.90% and the mAP of the bird’s eye view images is 28.60% respectively, which is quite a poor result. For the second model, the mAP of the front-view images detected by the second model is 10.13%, which is an utterly deplorable outcome. After transforming the images to the bird’s eye view, the mAP can reach 85.57%, achieving a significant improvement of at least 27.67% (57.90–85.57%), which shows that the proposed approach can be successful. The mAP of the third model testing the front-view image is 60.04%, while the mAP of the bird’s eye view image is 78.66%. From the previous results, it can be noticed that the same perspective model testing on the same perspective images can obtain a better performance, while the different perspective model testing on the different view achieves a much lower mAP. Therefore, the third model, which takes the front-view images and bird’s eye view images as input, plays an essential role. The model can detect the different perspectives of images, which makes the model more robust, general, and stable. The experiment has demonstrated that the perspective transformation is effective for object detection on the road surface.

Moreover, the experiment also compared the proposed method with other instance segmentation models, SOLO version 2 and YOLACT++ [29]. SOLOv2 is a box-free instance segmentation model using ResNet-101 as the backbone and FPN for multi-scale prediction. As for YOLACT++, it uses the architecture of RetinaNet combing with ResNet-101 and FPN. The result reflected in Table 5 indicates that the Mask R-CNN with the bird’s eye image significantly outperforms the other methods. Basically, the performance of using bird’s eye view images as input is better than the other two kinds of images as input. On the other hand, it is believed that the proposed method can work well after perspective transformation no matter which model is used.

#### 4.4.3. The Experiment Results of Data Augmentation

Data augmentation is a method that increases the amount of data in a limited dataset. Table 6 shows the comparative results of the model with data augmentation and without data augmentation. The mAPs of the first model testing on the front view and bird’s eye view are 58.27% and 33.96%, respectively, which increase from 0.37% to 5.36%. After the data augmentation is performed on the second model, there is no significant increase but there is a decrease of at least 5%. The mAP of the third model testing on the front-view images decrease by 2.33%, while testing on the bird’s eye view increase by less than 1%. It may be that there is no significant difference in the diversity of the data, so the detection does not improve enough. To summarize, in comparison with the model without data augmentation, the results can increase by 1–5% mAP, but there is no significant improvement. However, there is no denying that data augmentation is a good approach to augmenting the amount of data under limited resources.

#### 4.4.4. Examples of Object Detection Results

**Figure 10:**
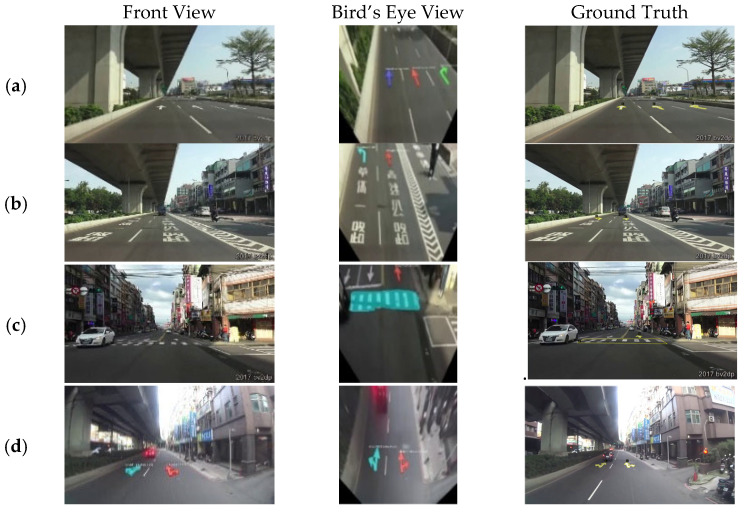
Examples of the bird’s eye view model testing on different cases.

Figure 10 shows the results of the front view and bird’s eye view in the same image, which uses the mixed bird’s eye view images as training input data. Some cases in the first column of Figure 10a–c are the detection results of the front-view images, which is detected background (false positive, FP), while the bird’s eye view images can be detected correctly. The object on the right-hand side in the first column of Figure 10d is incorrectly recognized as a special lane, while the bird’s eye view image is predicted correctly.

**Figure 11:**
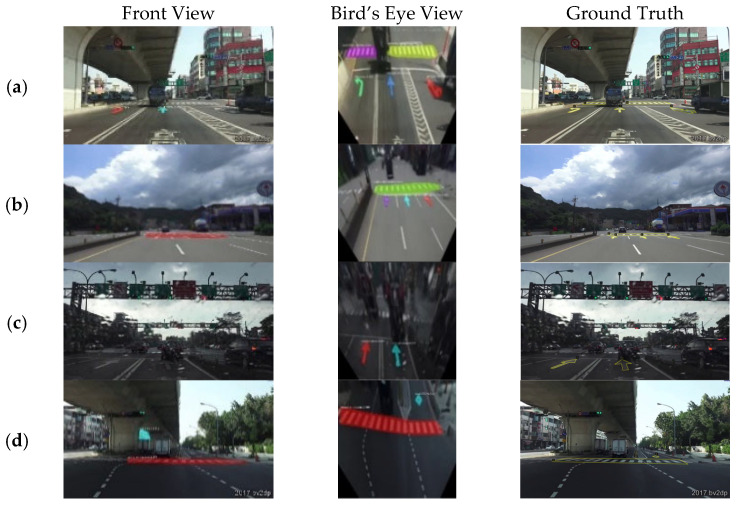
Examples of the front view with bird’s eye view model testing on different cases.

Figure 11 illustrates that some cases in the first row of the front-view images failed to detect the road markings at a far distance, such as pedestrian crossing (a), straight arrow (b,c), and special lane (d). However, after projecting to the bird’s eye view, the objects were accurately recognized even on a low-light rainy day (c). As displayed in Figure 10 and Figure 11, it is demonstrated that the proposed method can successfully detect small road markings at a great distance by converting the images into a bird’s eye view. In particular, the number of label samples after transforming will not change, so the comparison between the two types of images is based on the same foundation.

## 5. Conclusions

In this paper, the cross-field road markings have been successfully detected based on Inverse Perspective Mapping (IPM). After the perspective transformation, the distant objects on the road surface were detected, which solves the small object detection problem. First of all, the two open datasets derived from the virtual world and real world were mixed for the training data which can reduce the data preprocessing time and cost. The research, which trained three kinds of models according to the different perspective training images, presented different results. The testing phase, compared with the preliminary study, used front-view images to test on the road environment. IPM was performed on the input images to transform them into the bird’s eye view, which solves the “small objects at far distance” problem and the “perspective distortion of objects” problem, so that the model can clearly recognize objects on the road. Using three kinds of models to test the front-view images and bird’s eye view images can demonstrate the apparent result. In the second model test on the images after the IPM approach, it could reach an 85.57% mAP, which obtained the immense improvement of 27.67% (57.90–85.57%). The third model testing on the front-view images and bird’s eye view images also showed a remarkable improvement of accuracy by 18.62% (60.04–78.66%). Moreover, for the sake of making the model more robust and stable, the data augmentation method was employed to generate more data in the limited dataset. In comparison with the model without data augmentation, the result could increase by 1–2% mAP. We utilized Mask R-CNN as the implemented model and compared this with other models, SOLO and YOLACT, to ensure the proposed method could be realized successfully.

Much remains to be done for future work; it is also anticipated that the work can increase some different classes of road markings detection, such as “stop”, “slow”, “speed limit”, “bicycle sign”, “lane lines”, and so on, so it can produce a more reliable and stable detection model. In addition, the perspective transformation of the images is fulfilled by choosing four reliable points and then warping them into the 2D plane. The four points are decided depending on the different datasets to find the suitable points after removing the irrelevant background or to find the points along the roadside, so that the points follow the properties of the different datasets, lacking uniformity. If the dataset contains the camera parameters, the homography matrix is easily computed, but it is hard to ensure that all the datasets come with the camera information. Considering these limitations, the perspective transformation based on the deep-learning method can be examined and undertaken, which automatically produces the bird’s eye view images without manual selection and camera parameters. Moreover, other data augmentation techniques can be tried in the study to prove that the method is beneficial to augment the data. In spite of some limitations of the conclusion, the contributions of the study are seen to be compelling enough to encourage future investigation into both this and other road marking-related topics. We will also explore YOLO-based methods and state-of-the-art instance segmentation methods, such as YOLOv8, TSD-YOLO, and CBNetV2, to compare their performance with the approaches used in this paper. This will help evaluate the strengths of bounding box-level detection versus mask-level segmentation in road marking tasks. Additionally, an ablation study on data augmentation techniques, including brightness adjustment, contrast enhancement, and flipping, will be conducted to assess their individual contributions to model performance. Furthermore, we plan to design data augmentation strategies specifically tailored to road marking detection in diverse and complex environments such as wear and tear of road markings, and extreme lighting scenarios like glare or low-light conditions. These directions aim to build upon the current findings and further enhance the robustness and applicability of road marking detection methods.

## Figures and Tables

**Table 1:** Detailed specifications of the experimental environment.

Items	Specification
CPU	Intel i9-9900 3.5 GHz 10 cores
Memory	DDR4 2400 MHz 16 GB × 4
GPU	NVIDIA GeForce RTX2080 Ti
Operating System	Linux Ubuntu 18.04
Libraries	Python3.6, Tensorflow-gpu-1.12, CUDA 9.1

**Table 2:** Training parameters.

Items	Specification
Number classes	8 (including background)
Steps per epoch	Training samples/batch size
Epochs	100
Learning rate	0.001
Detection minimize confidence	0.9

**Table 3:** The comparison of the same-field and cross-field detection in the front view model.

Training Data	Same-Field	Cross-Field (Taiwan)
	Front	Front
Front View	98.22%	57.90%

**Table 4:** The comparison of the same-field and cross-field detection in the bird’s eye view model.

Training Data	Same-Field	Cross-Field (Taiwan)
	Bird’s Eye View	Bird’s Eye View
Bird’s eye view	97.02%	85.57%

**Table 5:** Comparative results of different models and our method.

Models	Training Data	Testing Data	
		Front View	Bird’s Eye View
Mask R-CNN	Front view	57.90%	28.60%
	BEV	10.13%	85.57%
	Front view and BEV	60.04%	78.66%
SOLO v2	Front view	15.32%	9.9%
	BEV	5.4%	42.60%
	Front view and BEV	23.60%	39.70%
YOLACT++	Front view	30.28%	9.64%
	BEV	28.84%	67.56%
	Front view and BEV	31.48%	64.76%

**Table 6:** Comparative results of the model with data augmentation and without data augmentation.

Training Data	w/o Data Augmentation		w/ Data Augmentation	
	Front View	BEV	Front View	BEV
Front view	57.90%	28.60%	58.27%	33.96%
BEV	10.13%	85.57%	10.85%	80.34%
Front view and BEV	60.04%	78.66%	57.71%	79.02%

## Data Availability

Data are contained within the article.
